# The impact of adopting stress‐tolerant maize on maize yield, maize income, and food security in Tanzania

**DOI:** 10.1002/fes3.313

**Published:** 2021-07-09

**Authors:** Girma Gezimu Gebre, Harriet Mawia, Dan Makumbi, Dil Bahadur Rahut

**Affiliations:** ^1^ Faculty of Environment, Gender and Development Studies College of Agriculture Hawassa University Hawassa Ethiopia; ^2^ International Maize and Wheat Improvement Center (CIMMYT Nairobi Kenya; ^3^ International Maize and Wheat Improvement Center (CIMMYT El Batán, Texcoco México; ^4^ Asian Development Bank Institute Tokyo Japan

**Keywords:** adoption, heterogeneity, income, productivity, stress‐tolerant maize varieties, Tanzania

## Abstract

Productivity growth emanating from scientific advances offered by biotechnology and other plant breeding initiatives offers great promise for meeting the growing food demand worldwide. This justifies investments in agricultural research and development that have led to the development of stress‐tolerant maize varieties (STMVs) in Africa. While most literature has documented the average impacts of STMVs on productivity, this paper is premised on the fact that benefits from technology adoption are not the same across household. The paper addresses this information gap by examining potential heterogeneity in yield, income, and food security benefits from of adopting STMVs using a sample of 720 maize‐producing households from Tanzania. The dose‐response continuous treatment effect method supported by an endogenous switching probit model was used to estimate the heterogenous impact of STMV adoption on the three outcomes of interest. Results show that, overall, the adoption of stress‐tolerant maize varieties increased maize grain yield by about 1 ton/ha, maize income by about $62/ha. The adoption of STMVs also reduced the propensity to report mild, moderate, and severe food insecurity by 34%, 17%, and 6%, respectively. There are substantial idiosyncratic variations in the productivity, income, and food security effects depending on the scale of adoption, with a higher impact at lower dose levels of adoption. The heterogenous and pro‐poor nature of STMV adoption is also revealed through nonparametric results showing higher productivity benefits among households that are less endowed with wealth and knowledge. These findings underscore the need for further scaling of stress‐tolerant maize varieties for greater impact on the livelihoods of poor small‐scale farmers in Tanzania.

## Graphical abstract

The article would be of great interest to the readers of the journal because it emphasizes on issues of scalability of climate smart adaptation strategies. It builds on previous literature that looks at the heterogeneity in the impact of adopting improved technologies but extends and applies it to stress‐tolerant maize in Tanzania. We can confirm that the material contained in the manuscript has not been published and is not being considered for publication elsewhere and does not infringe upon other copyright materials.

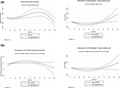

## 1 INTRODUCTION

Maize is the staple food for most Tanzanians and accounts for over 45% and 75% of the total cultivated land and cereal production, respectively. However, its productivity remains low, averaging about 1.4 MT/ha (FAO, 2014). The low yield performance of maize is due to the limited use of improved technologies (Takahashi et al., 2020). Moreover, the situation is exacerbated by the occurrence of abiotic and biotic stresses, such as drought and diseases, respectively, which also contribute to pre‐ and post‐harvest losses. As reported by de Janvry et al. (2001), the growth in agricultural production cannot come from area expansion since that has already become a minimal source of output growth at the world scale and negative source in India and Latin America. Thus, sustainable growth in the production will have to come from growth in yields emanating from scientific advances offered by biotechnology and other plant breeding initiatives. As such, a number (more than 70) of stress‐tolerant maize varieties (STMVs) with an enhanced ability to withstand abiotic stresses like drought have been developed, using innovative modern breeding technologies, to improve maize productivity. The STMVs have been developed over the years in a collaborative effort by the International Maize and Wheat Improvement Center (CIMMYT) and National Agricultural Research Organizations (NAROs), particularly in Africa (Fisher et al., 2015; Shiferaw et al., 2014; Simtowe, Marenya, et. al., 2019). In addition to stress and drought tolerance, the varieties often have other attractive traits, such as better responsiveness to inputs and good nitrogen use efficiency (Fisher et al., 2015). Moreover, STMVs has high resistance to low soil fertility, heat, disease such as maize lethal necrosis, and pests that affect maize productivity. They are well‐adapted to sub‐Saharan Africa and include hybrids and open‐pollinated varieties. STMVs are expected to increase maize yields by 20% to 30%, reduce yield variability, and reduce production risk (Simtowe et al., 2019). The STMVs are screened in each of the countries where they undergo extensive on‐station and multilocation on‐farm testing using participatory variety selection approaches with farmers across the different agro‐ecologies (Simtowe, Marenya, et al., 2019). The varieties that out‐yield popular commercial checks in those agro‐ecologies where they are tested are then selected for release and subsequent commercialization (Fischer & Hajdu, 2015; Fisher et al., 2015; Setimela et al., 2017). In Tanzania, such varieties are being produced and marketed to the farming communities by both smaller seed companies as well as multinationals.

This paper is motivated by the fact that although extant research has documented the average impact of adopting improved technologies on productivity and other outcomes, little attention has been paid to highlighting the fact that these effects are not the same across households. The first potential sources of heterogeneity, which is widely discussed in impact literature (Jaleta et al., 2018; Kassie et al., 2014, 2015) is the fact that adoption effects vary with the scale of the adoption because different levels of adoption scale are expected to trigger outcome‐responses of different magnitudes.

Second and an important source of heterogeneity in the impact relates to the fact that the first‐order effect, “the average impact of technology for adopters” is not static over time (de Janvry et al. 2001) for many reasons that include the learning effects and changing composition of adopters (more Schumpeterian farmers adopt first), among others (de Janvry et al. 2001).

Third, and a major motivation for this study is that the benefits from technology adoption may vary across farmers due to risk aversion. Related literature on the role of risk aversion on technology adoption and productivity (Feder, 1980; Lipton & Longhurst, 1989) shows how risk attitudes limit smallholder adoption of the new crop varieties and how this behavior is linked to a lack of an economic buffer, a key characteristic of poverty (Mosse, 2010; Fischer, 2016). This brings to question the widely acclaimed theory about the scale neutrality^1^ of seed technologies. In questioning the scale neutrality of seed technology, Fischer (2016) observes that (i) high yielding varieties are connected with other inputs, such as irrigation, that might not be equally divisible (see, e.g., Federet al., 1985, p. 280) and that (ii) the size of holding is a surrogate for a large number of factors such as access to credit, capacity to bear risks, access to scarce inputs (water, seed, fertilizers, insecticides), wealth, and access to information.

Therefore, rather than being a result of the introduction of a scale‐neutral crop technology, the scale‐neutral effects of the technology depended, in particular, on other investments in making the technology accessible and affordable to resource‐constrained farmers as well.

According to the risk aversion literature, it is argued that absolute risk aversion declines as wealth increases. The literature mentions subjective^2^ and objective risk^3^ by asserting that since smallholders are not as well supplied with agricultural advisory services and information on new products as large‐scale farmers, their behavior is more greatly affected by subjective risk (Feder, 1980; Fischer, 2016; Lipton, 1978). As a solution, Hazell (2009) expresses that investments in government extension services may reduce subjective risk and make the new crop technology useful for smallholders (Hazell, 2009). As observed by Tripp (2001), in Africa, there has been a continued decline in government funding for extension services, which increases smallholder exposure to subjective risk in relation to crop technology adoption. As such, there is a strong policy interest to understand the extent to which benefits from adopting improved technologies such as STMVs could vary with relative exposure to subjective risk among the farming households.

Moreover, environments inhabited by many smallholders increase objective risk and which forces poor farmers to be averse to such risks (Altieri et al., 2011; Dawson et al., 2008; Fischer, 2016). As such, poor households farm less optimal land characterized by infertile soils, deficient or excess rain which increases the (objective) production risk which can be reduced by stabilizing production through inputs such as irrigation and fertilizer (Darnhofer, 2010). Resource‐constrained smallholders are generally less able than large‐scale farmers to invest in agricultural inputs to stabilize production, they more frequently spread the risk by diversifying which affects their productivity and benefits from improved technologies.

Against this background, this paper seeks to contribute to the literature in two ways: first, we assess the average impact of adopting STMV on productivity, maize incomes, and food security. Second, beyond the average effects, the study aims to address the question of heterogeneity in the impact of STMV for two reasons: (i) seed technology interventions take the form of a continuous exposure such that farmers do not only adopt the new seed but adopt it at different intensities by allocating different amounts of land to the new seed technology. This provides ground for differences in responses to the different levels of adoption of the new variety. Hence, both the binary treatment status and level of treatment (or dose) become important in understanding the impact of the intervention. (ii), the adoption of improved seed requires complementary inputs and knowledge (proxies of risk exposure) and whose acquisition by the household might be influenced by the wealth and other access‐related factors, which have implications regarding how the innovations benefit each household. We, therefore, interrogate the questions: (i) to what extent is the impact of STMV adoption heterogenous? and (ii) to what extent is heterogeneity driven by objective and subject risk? We follow Baum and Cerulli (2016) to estimate a dose‐response function through a regression approach under the following assumptions: (i) exogenous continuous treatment (ii) treatment being endogenous and heterogenous to observable confounders. To the best of our knowledge, this is the first study to understand the potential sources of heterogenous impacts among maize‐based technologies. We follow Filmer and Pritchett (1998) to construct wealth and knowledge indices using weights chosen by principal components and use nonparametric techniques to compare the magnitude of benefits from the adoption of STMVs across farmers of different levels of wealth and knowledge endowment, which are also associated with levels of exposure to objective and subjective risks (Fischer, 2016), respectively. Section 2 provides a discussion of the empirical strategy, section 3 gives an overview of the survey design, data, and descriptive statistics. Section 4 presents and discusses the results, while conclusions and policy implications are presented in section 5.

## 2 CONCEPTUAL FRAMEWORK AND HYPOTHESES

### 2.1 Model

The interest of this study is twofold: (i) to assess how maize productivity, income, and food security situations respond to different intensities of the adoption of stress‐tolerant maize and (ii) whether such differential effects can partly be attributed to differences in levels of wealth and knowledge endowments. The first point, which is widely discussed in impact literature (Jaleta et al., 2018; Kassie et al., 2014) implies that going beyond a binary treatment case, the treatment effects are allowed to vary with the scale of adoption. Different levels of adoption scale are expected to trigger productivity and income responses of different magnitudes. The average treatment effect for a particular level on the scale of adoption is identified by comparing the maize productivity and maize incomes of households that planted stress‐tolerant maize to a given amount of land with households that did not plant stress‐tolerant maize.

The second point of interest in this paper relates to the fact that the benefits from technology adoption may vary across farmers due to risk aversion. Feder et al. (1985, p. 298). Arrow (1971) expresses that absolute risk aversion declines with increasing levels of wealth. Such literature argues that technology impacts are not scale neutral because risk‐averse farmers will adopt suboptimal amounts of the technology leading to lesser benefits than when the technology is adopted at optimal levels due to the lack of economic buffer. Fischer (2016) points out two forms of risk; namely subjective risk which relates to the lack of sufficient knowledge to assess a new technology and objective risk which relates to wealth with the poor having facing limited investment possibilities including farming in less optimal land characterized by infertile soils, poor rainfall while also facing difficulties to invest in agricultural inputs. This suggests that both forms of risks have an attenuation effect on technology benefits. These arguments are the basis of our key hypothesis in this paper that; although seed technology is divisible / scale‐neutral; its effects on the adopting households are determined by the household heterogeneity in both objective and subjective risk.

We follow Cerulli (2014) and Baum and Cerulli (2016) to employ the dose‐response model, which is an econometric model for estimating continuous treatment effects when responses are heterogeneous and where selection into treatment may be endogenous. The model is relevant to this study because a farmers’ decision to adopt stress‐tolerant maize may not be random and may be influenced by confounders and vice versa. The dose‐response treatment model under continuous treatment is chosen because as expressed by Cerulli and Poti (2014), it does not require full normality and it is applicable to our study where lots of the farmers have not yet started planting stress‐tolerant maize. Recently, dose‐response model was applied by Manda et al. (2020) to study the impacts of cowpea market participation on household food security and income in northern Nigeria.

Assuming two exclusive outcomes: *y*1*_i_* an outcome when a farmer *i* is an adopter of stress‐tolerant maize, and one $y0_{i}$ when the farmer does not adopt stress‐tolerant maize. The treatment indicator variable $w_{i}$ takes the value 1 when the farmer grows STMV and the value 0 when a farmer does not grow STMV. $x_{i}=\;{(x}_{1i}\cdots x_{\mathrm{Ki}})$ is assumed to be a row of vector of K exogenous and observable characteristics (confounders) for unit i=1,⋯,N. We let N be the number of farmers involved in the study, $N_{1}$ being the number of adopting farmers, and $N_{0}$ the number of non‐adopters with $N=N_{0}+N_{1}$.

The farmer i’s responses to the vector of confounding variables $x_{i}$ when the farmer adopts and does not adopt are expressed using functions $g_{1}\left(x_{i}\right)$ and $g_{0}\left(x_{i}\right)$, respectively.

We further follow Cerulli (2014) to assume $\mu_{1}$ and $\mu_{0}$ to be two scalars, and $e_{1}$ and $e_{0}$ as two random variables having zero unconditional mean and constant variance. Farmers are assumed to have different adoption intensities for stress‐tolerant maize $\left(t_{i}\right)\;$with non‐adopters having $t_{i}=0$ while adopters are assumed to take values greater than zero ($t_{i}>0$). $t_{i}$ is therefore assumed to take values strictly within the continuous range of [0:100] as the continuous‐treatment indicator, and ${h\;(t}_{i})$ as a general derivable function of ($t_{i}$).

Given the previous definitions, the potential outcomes are modeled in an additive form:

(1) $$\left\{\begin{array}{cc} y_{1}=\mu_{1}+g_{1}\left(x\right)+h\left(t\right)+e_{1} & \text{if}w=1\\ y_{0}=\mu_{0}+g_{0}\left(x\right)+e_{0} & \text{if}\;w=0 \end{array}\right.$$

where the h(t) function is different from zero only in the treated status:

(2) $$\left\{\begin{array}{cc} h(t)=0 & \text{if}\;w=0\\ h(t)\neq 0 & \text{if}\;w=1 \end{array}\right.$$

The treatment effect (TE) is defined as the difference $TE={(y}_{1}‐y_{0})$ and the population average treatment effects (ATEs) conditional on xand tdefined as:

(3) $$\begin{array}{c} ATE\left(x;t\right)=E\left(y_{1}‐y_{0}|x,t\right)\\ ATE\left(x;t>0\right)=E\left(y_{1}‐y_{0}|x,t>0\right)\\ ATE\left(x;t=0\right)=E\left(y_{1}‐y_{0}|x,t=0\right) \end{array}$$

where ATE indicates the overall average treatment effect, ATET indicates the average treatment effect on treated, and ATENT indicates the average treatment on untreated units (Cerulli, 2014). By the law of iterated expectation (LIE), we know that the population unconditional ATEs are obtained as:

(4) $$\begin{array}{c} ATE=E_{\left(x,t\right)}\left\{ATE\left(x;t\right)\right\}\\ ATET=E_{\left(x,t>0\right)}\left\{ATE\left(x;t>0\right)\right\}\\ ATENT=E_{\left(x,t=0\right)}\left\{ATE\left(x;t=0\right)\right\} \end{array}$$

where $E_{z}\left(.\right)$ identifies the mean operator taken over the support of a generic vector of variables z. By assuming a linear‐in parameters parametric form for $g_{0}\left(x\right)=x\delta_{0}$ and $g_{1}\left(x\right)=x\delta_{1}$ the ATE conditional on x and t becomes:

(5) ATE(x;t,w)=w.[μ+xδ+h(t)]+(1‐w).[μ+xδ]

where $\mu =(\mu_{1}‐\mu_{0})$ and $\delta =(\delta_{1}‐\delta_{0})$ and the unconditional ATE related to model (1) is equal to:

(6) $$ATW=p\left(w=1\right).\left(\mu +{\bar{x}}_{t>0}\delta +{\bar{h}}_{t>0}\right)+p\left(w=0\right).\left(\mu +{\bar{x}}_{t=0}\delta \right)$$

where $p\left(.\right)$ is a probability, and ${\bar{h}}_{t>0}$ is the average of the response function taken over t>0. Since, by LIE, we have that $ATE=p\left(w=1\right).\;ATET+p\left(w=0\right).\;ATENT$, we obtain from the previous formula that:

(7) $$\left\{\begin{array}{c} ATE=p\left(w=1\right)\left(\mu +{\bar{x}}_{t>0}\delta +{\bar{h}}_{t>0}\right)+p\left(w=0\right)\left(\mu +{\bar{x}}_{t=0}\delta \right)\\ ATET=\mu +{\bar{x}}_{t>0}\delta +{\bar{h}}_{t>0}\\ ATENT==\mu +{\bar{x}}_{t=0}\delta \end{array}\right.$$

where the *dose*‐*response* function is given by averaging $ATE\;\left(x,t\right)$over x **:**

(8) $$ATE\left(t\right)=\left\{\begin{array}{cc} ATET+(h\left(t\right)‐{\bar{h}}_{t>0}) & \text{if}\;t>0\\ \mathrm{ATENT} & \text{if}\;t=0 \end{array}\right.$$

The doss response is a function of the treatment scale or intensity t. The variable t also called the “dose” is generated by a procedure in SATA (treatment status‐0)/(r(max)0)*100.) such that it takes values in the [0;100] interval, where 0 is the treatment level of non‐treated units. The maximum dose is thus 100^4^.

The estimation of (8) under different identification hypothesizes is the main purpose of next sections.

### 2.2 Endogenous treatment estimation of the effect of adoption on productivity and income

The estimation of the causal parameters defined in (2) and (3) as well as the dose‐response function in (8) requires careful consideration. The first stage involves a consistent estimation of the “basic” parameters which are the parameters of the potential outcomes in (1). Substituting equation 1 into the potential outcome equation proposed by Rubin (1974) $y_{i}=y_{01}+w\left(y_{1i}‐y_{0i}\right)$ leads to the following baseline random coefficient regression:

(9) $$y_{i}=\mu_{0}+w_{i}.ATE+x_{i}\delta_{0}+w_{i}.\left(x_{i}‐\bar{x}\right)\delta +w_{i}.\left(h\left(t_{i}\right)‐\bar{h}\right)+\eta_{i}$$

where $\eta_{i}$=$e_{01}+w_{i}.\left(e_{1i}‐e_{0i}\right).$

We assume a parametric form of h(t)to estimate the basic parameters $\left(\mu_{0\;\;},\mu_{1}\;,\;\delta_{0},\;\delta_{1},\;ATE\right)$and all the remaining ATEs. When wand tare endogenous, unconfoundedness^5^ or conditional mean independence (CMI) hypothesis does is violated and hence the estimation of (10) by OLS becomes biased due to the failure in the orthogonality condition implied by Unconfoundedness as expressed by:

(10) $$E\left(\eta_{i}|w_{i},t_{i},x_{i}\right)=E\left(e_{1i}‐e_{0i}\right)|w_{i},t_{i},x_{i}\neq 0$$

Cerulli (2014) recommends the application of an Instrumental Variables (IV) estimation techniques to restore the consistency of parameter estimates. The model can, therefore, be presented in a structural form as:

(11) $$\left\{\begin{array}{c} y_{i}=\mu_{0}+x_{i}\delta_{0}+w_{i}.ATE+w_{i}\left[x_{i}‐\bar{x}\right]\delta +w_{i}T_{1i}+bw_{i}T_{2i}+cw_{i}T_{3i}+\eta_{i}\;\left(11.1\right)\\ w_{i}^{*}=x_{w,i}\beta_{w}+\varepsilon_{\mathrm{wi}}\;\left(11.2\right)\\ t_{i}^{\prime }=x_{t,i}\beta_{t}+\varepsilon_{\mathrm{ti}}\;\left(11.3\right) \end{array}\right.$$

where $T_{1i}=t_{i}‐E\left(t_{i}\right),\;T_{2i}=t_{i}^{2}‐E(t_{i}^{2}$), and $T_{3i}=t_{i}^{3}‐E\left(t_{i}^{3}\right);\;w_{i}^{*}$ represent the latent unobservable counterpart of $w_{i}$while $t_{i\;}$is only observed when $w_{i}=1$; $x_{\mathrm{wi}}$ and $x_{\mathrm{ti}}$ are sets of exogenous regressors, while $\varepsilon_{\mathrm{wi}}$, $\varepsilon_{\mathrm{ti}}$, and $\eta_{i}$ are corelated error terms.

Equations 11.2 and 11.3 are selection and treatment‐level equations, respectively. Equation 11.3 defines factors affecting the levels of treatment (level of STMV adoption) only for units that are eligible for the treatment. Equation 11.1, wand $T_{1},\;T_{2,\;}\;T_{3}$are endogenous hence the identification of linear system equation (11) requires the availability of two instrumental variables $z_{w,i}$and $z_{t,i}\;$which should be: (i) correlated with $w_{i}^{*}$and $t_{i}^{\prime }$, respectively; (ii) uncorrelated with$\;\varepsilon_{w,i}$, $\varepsilon_{t,i}$and $\eta_{i}$. The system (11) is estimated with a recognition that (11.2) and 11.3) are a bivariate sample selection model or type 2 tobit model (Heckman, 1979). Hence the estimation starts by jointly estimating equations 12.2 and 12.3 jointly by a type −2 tobit model, then computing the predicted values of $w_{i}\left({\bar{p}}_{\mathrm{wi}}\right)$ and $t_{i}\left({\bar{t}}_{\mathrm{wi}}\right)$ from the previous type 2 tobit estimation after which a two‐stage least squares (2SLS) is performed for equation 11 using IV techniques. Finally, the causal parameters of interest—ATEs and the dose‐res ponse function are estimated. Besides the dose‐response function and the other causal parameters of interest, the model allows also for calculating the average comparative response at different levels of treatment (as in Hirano & Imbens, 2004). The estimation is done using CTREATREG procedure in STATA.

### 2.3 Estimation of the impact of STMV adoption on food (in)security

The food (in)security in the paper is measured as a binary outcome variable. Some studies involving two binary outcomes have employed Heckman's two‐stage selection method to account for observed and unobserved heterogeneity between the adopters and non‐adopters (e.g., Kunstashula et al., 2014). However, Lokshin and Sajaia (2011) indicate that the two‐stage approach generates residuals that are heteroskedastic and require cumbersome adjustments to obtain consistent standard errors. Hence, we follow Lokshin and Sajaia (2011) by employing the endogenous switching probit (ESP) model to assess the impact of STMV adoption on food (in)security.

### 2.4 Estimation of the wealth and knowledge heterogeneity

The concern in this paper is on the differential impacts of adopting stress‐tolerant maize varieties based on wealth status which is related to objective risk aversion and based on the status of knowledge access which is linked to subjective risk aversion. Thus, the information about how farm households vary in terms of objective and subjective risk and the extent to which this relates to the technology adoption benefits is central to questions such as how to target and package supplementary support to farmers being targeted with the stress‐tolerant maize varieties. Given the focus of this paper, we apply the principal component analysis (PCA) to the survey data to generate two types of indices: the wealth index and the knowledge access index. This is consistent with some studies (Filmer & Pritchett, 2001; Gwatkin et al., 2000; McKenzie, 2003) which grouped household into predetermined categories, such as quintiles, reflecting different wealth levels.

The PCA multivariate statistical technique has been widely applied in the reduction of the number of variables in a data set into a smaller and more rational, and uncorrelated number of “dimensions” or principal components (Rao, 1964; Vyas & Kumaranayake, 2006). For example, for a set of variables $X_{1}$ through to $X_{n}$,

$$PC_{1}=a_{11}X_{1}+a_{12}X_{2}+\cdots +a_{1n}X_{n}$$

$$PC_{m}=a_{m1}X_{1}+a_{m2}X_{2}+\cdots a_{\mathrm{mn}}X_{n}$$

Where $a_{\mathrm{mn}}$ represents the weight of the *m*th principal component and the *n*th variable. The weights for each principle principal component are given by the eigenvectors of the correlation matrix, or if the original data were standardized, the co‐variance matrix (Vyas & Kumaranayake, 2006).

#### 2.4.1 Selection of Wealth‐related variables

We followed McKenzie (2003) to measure the wealth status of the household by selecting, a set of asset variables such that it was broad enough to avoid problems of “clumping” and “truncation”. The variables included the number of rooms for the house, cemented house floor, ownership of flush toilet, radio, television, watch/clock, mobile phone, computer, and mattress, ownership of livestock such as cattle, goats, and poultry, use of electricity for cooking, and farm size.

#### 2.4.2 Selection of knowledge access‐related variables

In order to measure the knowledge access status, of the household, the following variables were selected: whether one received information about improved technologies, years of education of the head of household, years of farming experience, aware of stress‐tolerant maize varieties, whether received rainfall forecast information, membership in a group, and frequency of contacts with an extension agent.

#### 2.4.3 Application of PCA and construction of wealth and knowledge scores

The PCA analysis was conducted using STATA by deriving the eigenvectors from their correlation matrix because we did not standardize our data and the therefore the data were not expressed in the same units to ensure that all data have equal weight. While we allowed for the extraction of four principal components, we restrict our interpretation of the wealth and knowledge indices to the first principal component. The wealth‐ and knowledge‐related variables (indices) with mean equal to zero and a standard deviation equal to one were constructed by using factor scores from the principal component as weight. Households were later classified into wealth and knowledge groups (quintiles) using the constructed household wealth and knowledge scores, respectively. The differential impact of adopting stress‐tolerant maize was identified by non‐parametrically comparing the average impact across the different quintiles.

#### 2.4.4 Definition of treatment and outcome variables

The treatment variable of interest is the extent of adopting STMVs. Definition for adoption varies across studies (Doss, 2006). The appropriates of each definition depend on the specific context (Gebre et al., 2019). In this paper, we define adoption as the planting at least one STMV in a plot of maize. A plot‐level analysis indicates that about 51% of the maize plots were planted with STMVs^6^. The interest of this paper is to explore whether or not the benefits from adopting STMV vary with the scale of cultivation of STMV. The extent of cultivating STMV was captured as land in hectares planted with STMVs. However, Cerulli (2015) indicates that if the continuous treatment variable does not naturally lie between 0 and 100, or if it does not meet normality assumptions in its distribution, one can use a suitable transformation to ensure that it falls within this interval and that it is normally distributed. Although the actual size of the land allocated to STMVs falls between 0 and 100 ha, it does not have a normal distribution hence in the empirical estimation the variable was transformed accordingly following Cerulli (2015).

The first outcome variable is the yield of maize measure in kg/ha. This was measured by collecting information on the quantity of maize harvested for each of the plots cultivated. We seek to understand the average and heterogenous yield effect of growing STMV.

The second outcome variable of interest is the maize income or the maize gross margins. This was computed by deducting the total variable cost of producing maize from the total monetary value^7^ of maize produced for each of the maize plots. The interest is to understand the average and heterogenous income effect of adopting STMVs.

The third outcome variable of interest is the food security status of a household which was measured by constructing a Household Food Insecurity Access Scale (HFIAS) as a measure of the prevalence of household food insecurity. The HFIAS was developed by the United States Agency for International Development (USAID) Food and Nutrition Technical Assistant (FANTA) project. It is based on the assumption that the experience of food insecurity (access) causes predictable reactions and responses that can be captured and quantified through a survey and summarized on a scale (Coates et al., 2007; Headey & Ecker, 2012). In the HFIAS approach, respondents are asked nine questions related to the occurrence of food insecurity and frequency‐of‐occurrence over a four‐week recall period. The occurrence questions represent an increasing level of severity of food insecurity, and “frequency‐of‐occurrence” questions are asked as a follow‐up to each occurrence question to determine how often the condition occurred. That is if the respondent answers “yes” to an occurrence question, a frequency‐of‐occurrence question is asked to determine whether the condition happened rarely (once or twice), sometimes (three to ten times), or often (more than ten times) in the past four weeks (30 days) (Chinnakali et al., 2014; Coates et al., 2007). The HFIAS approach yields information on food insecurity (food access) at the household level.

## 3 SURVEY DESIGN AND DATA

**FIGURE 1:**
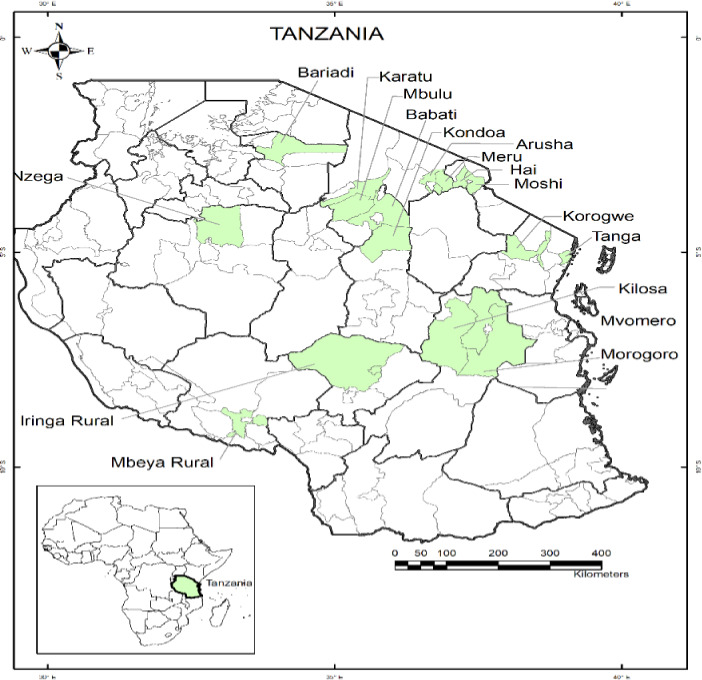
Map showing survey sites in Tanzania

The study is based on the household survey data collected in December 2018 and covering 720 households in 39 villages across 17 districts and 10 regions of Tanzania (see Figure 1). Surveyed areas included: Morogoro region (Kilosa, Morogoro rural and Mvomero districts); Iringa region (Iringa district); Mbeya region (Mbeya district); Tabora region (Nzega district); Manyara (Mbulu and Babati districts); Simiyu region (Bariadi district); Tanga region (Korogwe and Tanga districts); Dodoma region (Kondoa District); Arusha region (Karatu, Meru and Arusha Districts); and the Kilimanjaro region (Moshi rural and Hai districts). A three‐stage sampling technique was used, combining purposive and random sampling. The first stage selected regions, followed by the selection of villages, using probability proportional to size sample design. The third stage involved a random sampling of households within each village.

A semi‐structured questionnaire was designed and tested to collect a range of information related to household demography, socioeconomic, and agronomic features, maize production, and food security, including perceptions of household food security status. The questionnaire also captured some individual, household, and plot‐level attributes (e.g., plot‐level maize yield and maize income), as well as institutional arrangements besetting household on‐farm management.

## 4 RESULTS AND DISCUSSION

### 4.1 Descriptive statistics from household survey

The variables used in the analysis are disaggregated by the STMV adoption status of the households, and descriptive statistics are presented in Table 1.

**TABLE 1:** Descriptive statistics for Maize farmers in Tanzania

Characteristic	Full sample (*n* = 720)	Adopters (*n* = 339)	Non‐adopters (*n* = 381)	Difference
Dependent variables				
Average STMV plot size (ha)	0.42	0.90	0.00	0.90***
Yield (kg/ha)	1537	1672	1408	264***
Income (USD/ha)	137.81	139.27	136.27	3.00
Food security status (%)				
Food secure	0.483 (0.50)	0.496 (0.50)	0.480 (0.50)	0.016 (0.037)
Mild food insecurity	0.218 (0.411)	0.281 (0.428)	0.191 (0.394)	0.090 (0.030)
Moderate food insecurity	0.241 (0.417)	0.197 (0.217)	0.251 (0.42)	−0.054* (0.031)
Severe food insecurity	0.058 (0.226)	0.026 (0.169)	0.078 (0.269)	−0.052*** (0.016)
Independent variables				
Household size (No.)	5.86	5.91	5.82	0.09
Gender (% male)	87.13	89.22	84.93	4.29*
Age (ears)	52.45	51.44	52.56	−1.12
Education (years)	6.39	7.00	6.00	0.100***
Total farm size (ha)	3.50	3.30	3.60	−0.28
Total maize farm size (ha)	1.74	1.71	1.75	−0.04
Received seed information (%)	21.4	23.9	18.8	5.10*
Practicing intercropping (%)	44	45	42	3.00
In groups as members (%)	64	70	57	13.00***
Received information on expected rainfall patterns (%)	21	24	19	5.00*
Chemical fertilizer kg/ha	52	55	49	7.00*
Organic fertilizer kg/ha	34	34	35	−1.00
Credit access (%)	28.72	31.61	26.1	5.40*
Pesticides (Lit/ha)	1.20	1.20	1.10	0.01
Herbicide use (Lit/ha)	0.17	0.11	0.25	−0.14
No soil erosion (%)	54.60	52.7	56.50	−3.90
Marital status (% married)	81	84.50	77.30	7.20**
Agriculture self‐employed (%)	93.50	94.10	92.80	1.30

*, **, *** Significant at 10%, 5%, and 1%, respectively.

#### 4.1.1 Outcome variables

The average yield of maize for the whole sample was 1537 kg/ha with adopters registering significantly higher yields (1672 kg/ha) than non‐adopters (1408 kg/ha). The average maize income for the sample was US$138/ha and there was no significant difference in the maize income between adopters and non‐adopters. The results show that 48% of the households were food secure, while 22% and 24% were mildly food insecure and moderately food insecure, respectively. The rest 6% were severely food insecure in the study areas in Tanzania.

#### 4.1.2 Independent variables

Several independent variables were considered in this analysis. With regard to the gender of the head of household, about 87% of the households were male‐headed, and there was no difference in the proportion of male‐headed households between adopters and non‐adopters. The average household size was 5.9 persons, with no statistical difference between adopters and non‐adopters. Most of those interviewed were middle‐aged farmers as they had an average of 52 years of age with an average land holding size of 3.5 ha. About 21% of the households reported receiving information about new maize varieties in 2018, with a significantly higher proportion of adopters (24%) acknowledging receipt of such information than non‐adopters (19%). As regards the information sources, other farmers, field day demonstrations, and the government were the most preferred information sources about the new maize varieties. Membership in social groupings such as cooperatives and farmer groups, and faith‐based organizations can have a significant impact on adoption (Bandiera & Rasul, 2006). In our survey, membership in farmer groups was quite high and reported by 64% of the respondents, with adopters reporting significantly higher levels of membership (70%) than non‐adopters (57%). The data revealed a statistical difference in years of education between the adopters and non‐adopters of STMVs. The adopters had a mean of 7 years of education while non‐adopters had an average of 6 years.

The use of inorganic fertilizers was reported by 52% of the households and it was more prevalent among adopters. Access to credit is critical for the purchase of inputs. In this study, 29% of the households obtained credit, with a significantly higher proportion of adopters (32%) than non‐adopters (26%) reporting receiving credit in the 2018 cropping season.

### 4.2 Stochastic dominance analysis

**FIGURE 2:**
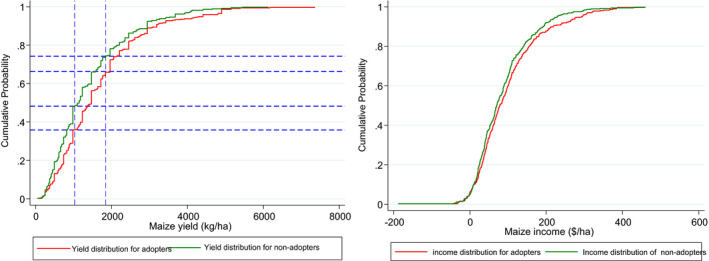
Cumulative density function of maize yields and incomes for adopter and non‐adopters of STMVs

In order to examine the yield and income differences of STMV adopters and non‐adopter, a stochastic dominance analysis was performed. As depicted in Figure 2, the cumulative distribution functions (CDFs) for the maize yields and incomes of adopters of stress‐tolerant maize dominate those of the non‐adopters. Kolmogorov–Smirnov test for first‐order stochastic dominance test also shows that the CDFs of adopters of STMVs stochastically dominate that of the non‐adopters for both maize yield (*p *= 001) and net maize income (*p *= 001). Results show that, for yield, more non‐adopters (48%) obtained maize yields less than 1000 kg/ha compared to 35% who got the same yield among adopters. Similarly, more adopters (34%) reported maize yields of more than 2000 kg/ha than non‐adopters (26%). However, these distributional differences in maize yields and incomes might be influenced by many factors, and the interest in this study to examine the extent to which these differences can be explained by features inherent in the STMVs particularly because improved technology comes with needs for improved inputs or by features inherent in STMVs that remain unaffected by context. Moreover, it is the interest of this paper to understand if both yields and incomes vary depending on the wealth status of a household and whether indeed the yield and income benefits from adopting STMV also vary with the wealth status of a household.

### 4.3 Observed maize yield differences by wealth and knowledge endowments

Yield differentials without controlling for observable and unobservable factors were examined between different classes of wealth and knowledge endowments. As depicted in Table 2, observed average maize yields vary substantially across wealth groups. The average yield for the poorest group (the 1st quantile) is just 1236 kg/ha compared to 1891 kg/ha for the wealthiest group (5^th^ quantile) representing a 52% yield difference. The higher yields among the wealthier households can be explained by the fact that apart from the seed technology, maize productivity is largely driven by other inputs such as fertilizer and other observable factors that wealthier households are more likely to access than their poor counterparts. This is apparently consistent with the fact that wealth is a surrogate for a larger number of potentially important factors such as access to credit, capacity to bear risk, access to scarce inputs (water seed fertilizer, insecticides), and access to information (Feder et al., 1985).

**TABLE 2:** Observed maize yield patterns by wealth group and adoption status

	Yield (kg/ha)			
Wealth group (1 = less wealthy; 5 = most wealthy)	Adopters	Non‐adopters	All	Difference
1st quantile	1352	1174	1236	177
2nd quantile	1581	1319.	1445	261*
3rd quantile	1836	1201	1479	635***
4th quantile	1541	1786	1646	−245
5th quantile	1950	1804	1891	146

*, **, *** Significant at 10%, 5%, and 1%, respectively.

Similarly, maize yields vary substantially across knowledge endowment groups (Table 3). The average yield for the least endowed group (the 1st quantile) is just 1209 kg/ha compared to 1893kg/ha for the most endowed group (5th quantile). The higher yields among the more knowledge endowed groups can be explained by the fact that knowledge enhances good agronomy and reduces exposure to subjective risk, leading to improved investment and improved economic efficiency. Apparently significant differences in yields between adopters and non‐adopters are mainly observed in the first quantile of the knowledge endowment and in the second and third quantiles of the wealth endowment, with non‐adopters reporting significantly lower yields than adopters. The observed yield differences between adopters and non‐adopters fizzle out with increasing wealth and knowledge endowment.

**TABLE 3:** Observed maize yield patterns by knowledge endowment and adoption status

Knowledge group (1 = less endowed; 5 = Most endowed)	Yield (kg/ha)			
	Adopters	Non‐adopters	All	Difference
1st quantile	1462	1092	1209	369***
2nd quantile	1491	1413	1449	78
3rd quantile	1515	1405	1448	110
4th quantile	1784	1562	1687	221
5th quantile	1890	1901	1893	−11

*, **, *** Significant at 10%, 5%, and 1%, respectively.

**FIGURE 3:**
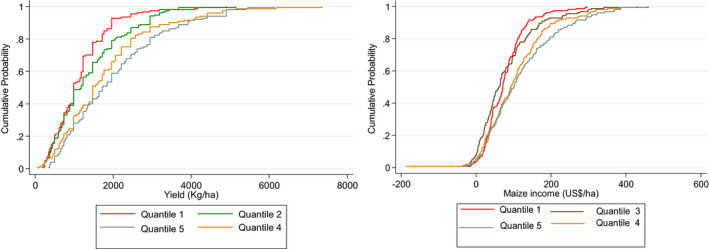
CDF of maize yields and incomes by level endowment

Figure 3 depicts cumulative distribution functions (CDFs) for the maize yields and incomes for different wealth endowment categories. The results show that both yields and incomes of wealthier groups of farmers dominate those of the poor farmers. Thus, the cumulative distribution functions (CDFs) for the maize yields and maize incomes of wealthier households dominate those of poor households.

### 4.4 The impacts of STMV adoption on yield, incomes, and food security

#### 4.4.1 The impact on maize yields

To measure the potential benefits from adopting STMVs, we estimate plot‐level maize yield and income response functions to the amount of land planted with STMV using the continuous treatment effect approach. The empirical results, after controlling for observable and unobservable factors, are presented in Table 4. The estimates are based on the instrumental variable approach^8^ where we instrument the household‐level treatment with two separate exclusion restrictions and hence, they do not suffer from endogeneity bias. The first stage regression estimates of these models are reported in Tables A1 and A2 in Appendix. Both instrumental variables (exposure to STMV, and the land holding size per household) were found to be relevant instruments and they are statistically significantly (at the one percent level) associated with STMV adoption and they were insignificant in the yield and income equations.

**TABLE 4:** Productivity and income impact of adopting STMVs

	A Maize yield (Kg per ha)		B Maize income (US$ per ha)	
	1	2	3	4
Variables	Coef	SE	Coef	SE
Log of maize area (treatment)	1,038.0***	351.4	61.640**	28.252
Seed (Kg/Ha)	337.521***	96.405	17.455**	7.751
Ln Fertilizer (Kg/Ha)	122.285***	23.18	5.515***	1.864
Sq. Fertilizer (Kg/Ha)	21.867**	10.659	−0.181	0.857
Pesticide (Lit/Ha)	10.377	11.586	−0.922	0.932
Herbicides (Lit/Ha)	35.833	22.168	1.69	1.782
Organic fertilizer Kg/Ha	17.822**	8.241	1.742***	0.663
Soil Fertility (1= Fertile soils)	172.856**	85.514	12.323*	6.876
Farms with no soil erosion	189.356**	79.347	19.528***	6.38
Farms with cover crop remains	169.694	122.727	17.299*	9.868
Rented land (Ha)	−54.321	112.582	−6.299	9.052
Household size (Number)	155.591*	87.264	14.687**	7.016
Households able to build savings	524.787***	141.097	37.773***	11.345
Households with little savings	−26.812	87.746	−5.875	7.055
Group membership (1=Yes; 0=Not)	2.026	88.921	−2.562	7.15
Fertilizer heterogeneity (ws_lnfertha)	−75.899*	45.755	−5.183	3.679
Morogoro site	289.247*	165.685	16.282	13.322
Iringa site	402.683**	185.916	36.547**	14.948
Mbeya site	610.068***	169.377	39.516***	13.618
Tabora site	224.953	146.916	25.432**	11.812
Manyara site	751.442***	148.878	52.225***	11.97
Simiyu site	386.216*	217.256	29.082*	17.468
Kilimanjaro site	443.274***	160.586	38.064***	12.912
Tw_1	−268.791**	104.895	−15.971*	8.434
Tw_2	10.991**	4.523	0.603*	0.364
Tw_3	−0.089**	0.037	−0.005	0.003
Constant	−486.825	391.566	−12.391	31.483
Observations	768		768	
*R* ^2^	0.264		0.114	

*, **, *** Significant at 10%, 5%, and 1%, respectively.

The estimated coefficient for adoption of stress‐tolerant maize in the model specifications is an estimate of the average treatment effect, ceteris paribus, of adopting STMV on maize yield and maize income. The coefficient of STMV adoption is significant and positive. Results imply that, on average and after controlling for observable and unobservable factors, STMV adoption increases maize yields by 1038 kg/ha. This finding is consistent with other studies on drought‐tolerant maize varieties, such as Simtowe, Amondo, et al. (2019) and Amondo et al. (2018); however, the relatively higher impact in this study could be attributed to the fact that in Tanzania, a larger proportion of farmers still grow unimproved and composite maize varieties. Other significant and positive predictors from the extended specifications are log of seed per hectare, log of inorganic fertilizer applied per hectare, log of organic fertilizer applied. A higher intensity of these inputs applied to the maize crop increases productivity. The initial soil conditions such as the perceived soil fertility condition of the plots where maize is grown also affect productivity with soils perceived to be fertile to positively influence productivity. Moreover, soils perceived to be less eroded also improved maize productivity. Intercropping maize with cover crops also increased maize productivity. Other location dummies such as Iringa, Mbeya, Manyara, and Kilimanjaro also positively and significantly influenced maize yield which suggests that there are other factors in these regions, perhaps not captured in this study, that make them agro‐ecologically better suited for the productivity of maize. The interaction term between fertilizer and adoption was positive and statistically significant in the yield specification a finding that suggests that not only does the adoption of STMVs benefit farmers but that it also positively influences farmers to use more fertilizer which jointly increases maize yields.

#### 4.4.2 The impact on maize income

The results of the impact of STMV adoption on maize income per hectare are presented in Table 4. Results indicate that STMV adoption has a significant positive effect on maize income. On average, an adopter gets US$62/ha more than a non‐adopter. Moreover, the interaction term between fertilizer use and adoption in the income specification was not significant, a finding suggesting that although adoption increases income, its income effect is the same for those who use and those who do not use inorganic fertilizer. Other factors that positively increased maize incomes included seed density, fertilizer, use of organic fertilizer, growing maize on fertile soils, farm plots with less soil erosion, larger households, and households with higher incomes. As in the case of maize yields, other location dummies such as Iringa, Mbeya, Manyara, and Kilimanjaro also positively and significantly influenced maize incomes.

### 4.5 Heterogenous impacts and dose‐response functions

**FIGURE 4:**
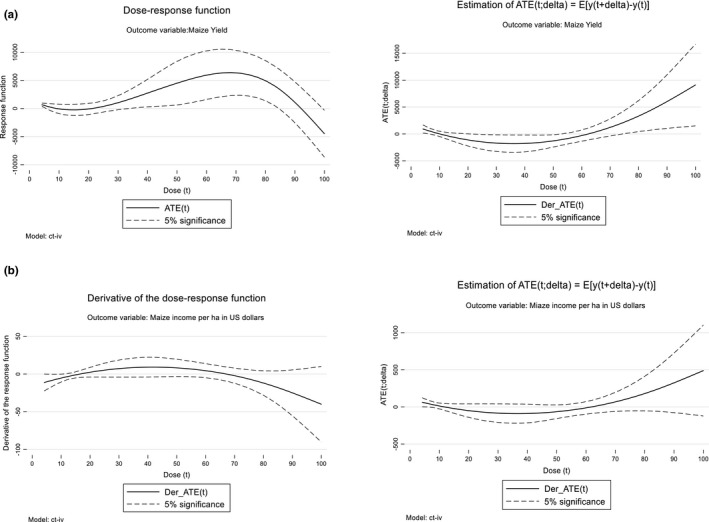
Distribution of the dose response functions and derivatives for yield and income

Households have different scales of STMV adoption (dose) which may have different impacts on maize productivity and incomes. Following Cerulli (2014) we apply the continuous treatment effects procedure in STATA to analyze the impact of different levels of STMV adoption intensities on maize yields and income. Based on the regression results in Table 4, we determine the average expected conditional yield and income effects and estimate dose‐response functions (DRF). Figure 4a depicts the DRF in which the impact of different STMV adoption scales on maize yield is assessed. The figure shows an inverted U relationship between the yield increase and STMV adoption scale or intensity. The inverted U relationship implies that yield among STMV adopters increases at lower adoption scale and reaches the peak before falling as the dose level increases. In other words, the adoption of STMV positively impacts yield growth at lower levels of adoption scale, reaches maximum and falls with increased adoption scale thereby exhibiting diminishing returns. At a dose level of about 70, households attain the highest yield increase of about 6000kg/ha but this reduces to zero at a dose level of 93 and to a huge yield reduction effect of at a dose level of 00. A similar distribution is observed for the DRF for maize income (Figure 4b). These findings provide extra insights on the yield and income benefits of adopting STMVs indicating that although the average treatment effect (ATE) is positive, the dose response suggests heterogenous effects over the adoption scale and with negative yield and income effects at highest levels of adoption scale.

### 4.6 Risk aversion as a potential source of impact heterogeneity

**FIGURE 5:**
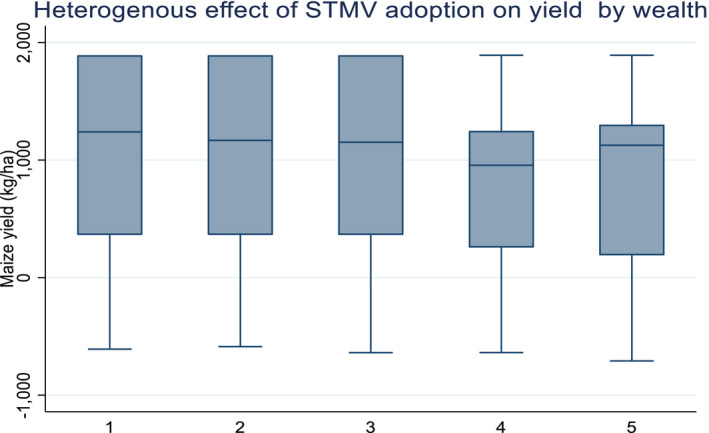
Yield effects of adopting STMV by wealth group

We also seek to understand whether the effects of adopting STMV vary depending on risk behavior proxied by the wealth status of the household. The role of objective risk as a source of heterogeneity on maize productivity and in income benefits accruing from adopting STMVs is assessed by comparing these benefits across households of different wealth categories. We compute the relative wealth and knowledge index of farmers using the principal component analysis and examine differences in yield and income effects of adopting STMVs across households belonging to different wealth and knowledge quintiles. As depicted in Figure 5 the yield effects of adopting STMVs was highest in the first quantile (poorest group), and lowest in the last two quantiles (wealthiest groups). These findings appear to be inconsistent with observed yield differences (before controlling for observable and unobservable factors) between wealthier and poor groups. They suggest that, after controlling for observable and unobservable factors, poor farmers stand to benefit more from adopting STMVs than their well‐off counterparts. On the centrally, literature on risk aversion, largely suggests higher expected benefits among wealthier and more knowledgeable farming groups resulting from increased use of complementary inputs which the poor may not afford. The findings are entirely surprising and quite intuitive in that when compared within each wealth category, improved seed is a big game‐changer for the poor adopting households when compared to the poor non‐adopting households. Thus, although in absolute terms the observed average yields among the poor households (both adopters and non‐adopters) are much lower (Table 3), they benefit the most from adopting STMVs. The declining yield benefits in the wealthier category can best be explained by understanding the differential application of other complementary variables that have a confounding effect on yield such as fertilizer. The wealthier non‐adopting households may be compensating the non‐adoption of improved seed with better husbandry practices not captured in this study and which reduces the yield gap between them and their adopting wealthier counterparts.

**FIGURE 6:**
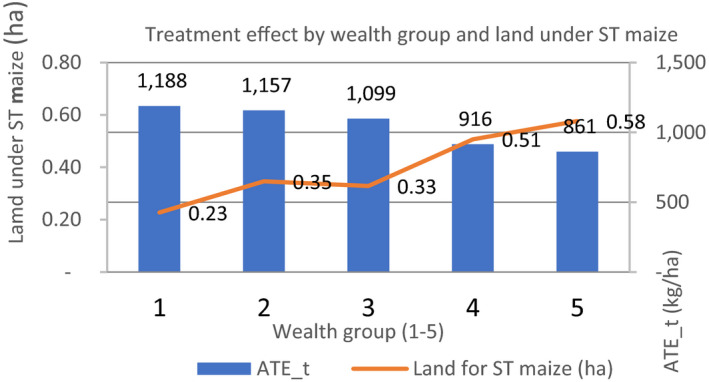
Yield effects by wealth and STMV land (ha)

It can also be understood that from Figure 6, poor households allocate less land to STMV, averaging 0.23ha than wealthier households who allocate about twice the size of land (0.58ha) to STMV. There is, therefore, temptation to attribute the high yield benefits among the poor to inverse plot‐size productivity relationship (IR) hypothesis which states that land productivity decreases as plot size increases (Desiere & Jollife, 2018). However, this is not the case in this study because the observed yield for the poor households was lower than those of the wealthier counterparts (Table 3). Moreover, the observed yield difference between adopters and non‐adopters was also only significantly different among the poor households, a fact than reinforces our earlier argument of the pro‐poor nature of improved seed technology. Collier and Dercon (2014) and Gollin (2018) argue that small farms in developing countries can be productive but not in the sense of technological productiveness but because of the imperfection in factor (e.g., labor) markets.

The higher yield benefits observed among households cultivating less STMV are also consistent with the results from the dose‐response function which show much higher benefits at a lower scale of adoption and declining benefits at higher adoption scale.

### 4.7 The impact of STMV adoption on food security

There are significant differences in the food insecurity status between STMV adopters and non‐adopters in Table 1; however, those differences did not take into account selectivity bias due to observable and unobservable factors. Hence, Table 5 presents endogenous switching probit (ESP) estimates of the effects of adopting STMVs on food security status that controls for both observable and unobservable factors. The results show that adopting STMVs increases the probability of a household to be food secure by 37.8%. The adoption of STMV also reduces the probability of reporting mild food insecurity, moderate food insecurity, and severe food insecurity by 34.1%, 16.5%, and 6.2%, respectively. These findings are unsurprising given the average maize yield gains of about one tone per hectare for STMV adopting households. These food security benefits from adopting STMVs are consistent with those observed by Jaleta et al. (2018) who report positive effect of improved maize adoption on food consumption among maize‐producing households in Ethiopia and also in line with the results of Manda et al. (2018) who reported a significant impact of improved maize variety on the food security status of farm households in Eastern Zambia.

**TABLE 5:** ESP estimates of the impact of adopting STMV on food (in) security status

Outcome variables	Average treatment effect (ATT, ATU, ATE)					
	ATT		ATU		ATE	
	Coef	SE	Coef	SE	Coef	SE
Food secure	0.463***	0.012	0.301***	0.007	0.378***	0.005
Mild food insecure	−0.733***	0.008	0.018*	0.008	−0.341***	0.007
Moderate food insecure	−0.322**	0.008	−0.020*	.009	−0.165**	0.005
Combined Severe and moderate food insecurity	−0.766***	0.010	0.578**	0.011	−0.062***	0.008

**FIGURE 7:**
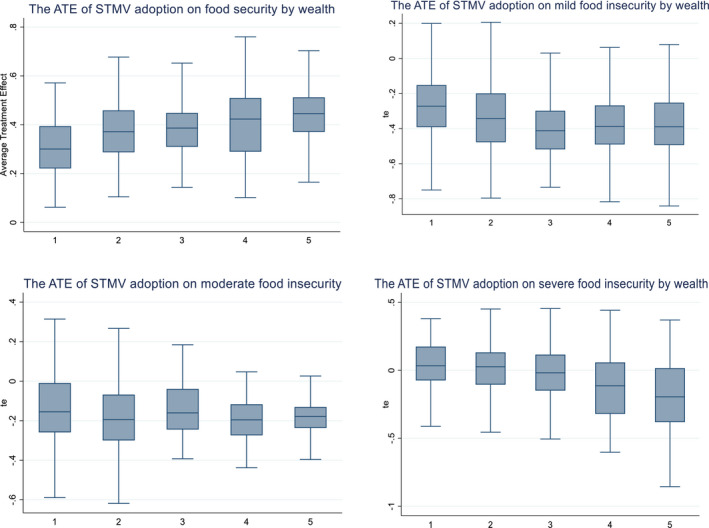
ATE of STMV adoption on food (in)security status by wealth

Since impact heterogeneity was one of the major interests in the paper, the food (in) security effects of adopting STMVs were further disaggregated wealth endowment category of the households. As depicted in Figure 7, results indicate food security effects vary substantially across different wealth classes of households and depending on the scale of food insecurity. The effect of improving food security is higher among wealthier households than their poor counterparts. The effects on reducing mild, moderate, and severe food insecurity are also higher among wealthier households and low among poor households. The findings are quite intuitive in that although poor households registered higher yield benefits, the absolute quantities produced are quite low due to smaller land holdings. The poorest group of households only allocated 0.23ha of land to STMVs, which may not be enough to produce adequate maize to feed a household of about six persons for the full year.

## 5 CONCLUSION AND IMPLICATIONS

In this study, we examined the impact of STMV adoption on productivity, income, and food security and their variability across different classes of wealth using survey data from Tanzania. With regard to the impact on productivity and incomes, we employed the dose‐response model, which is an econometric model for estimating continuous treatment effects when responses are heterogeneous and where selection into treatment may be endogenous. The impact on food (in) security was examined by employing the endogenous switching probit (ESP) model. The results show that the adoption of STMVs significantly increased productivity and maize incomes. The adoption of STMVs is also found to positively influence food security while reducing the probability of reporting, mild, moderate, and severe food insecurity. Our results also show that inorganic and organic fertilizers and seeds positively influence maize yields. Other important determinants of productivity with a positive effect include initial soil fertility levels, soil erosion, and the intercropping with cover crops. While the STMV adoption effects for both yield and income are positive, they become negative only above an adoption dose level of 90, suggesting the possible presence of an optimal size of continuous treatment for the average land allocated to STMVs among adopters beyond which adoption does not benefit the household. This suggests that as households increase the amount of land under STMV to a certain level, they fail to increase the use of other complementary inputs required to support crop growth such as fertilizer and the appropriate agronomy leading to diminishing returns. The results stand up to a wide range of diagnostic checks and are confirmed in the heterogeneity analysis. The findings are in tandem with findings by Simtowe, Amondo, et al. (2019); and Amondo et al. (2018), which suggests that scaling the cultivation of STMVs has great potential for transforming the maize sector in Africa. This study also highlights the importance of capturing the heterogenous impacts of adoption. We show a positive interaction between STMV adoption and fertilizer application demonstrating the positive effects of STMV adoption in improving the maize productivity among households that apply fertilizer to maize. The pro‐poor nature of STMV adoption is demonstrated by the higher productivity impacts attained by the poorest group of households, providing justification for policy initiatives that target poor households with smaller holding as beneficiaries of improved seed.

## ENDNOTES

^1^
One of the reasons being that crop technology is divisible and has equal benefits to farmers regardless of their size of land on which the new technology is planted

^2^
Subjective risk relates to not having sufficient knowledge to assess a new technology

^3^
Objective risk relates to the direct link between risk aversion and wealth as suggested by Feder et al., (1985). The poor have limited economic margins to cope with various uncertainties and limited possibilities to make investments to improve their situation

^4^
Cerulli (2015) indicates that if the continuous treatment variable does not naturally lie between 0 and 100, one can use a suitable transformation to ensure that it falls within this interval.

^5^
According to Rubin (1974), unconfoundedness states that conditional on the knowledge of the true exogenous confounders x, the conditions for randomization are restored and causal parameters become identifiable.

^6^
There are several drivers to adoption, but clearly, in seed‐related technologies, as the case of this study area; two key drivers are of consideration. First, a household cannot adopt STMVs if they are not aware of their existence. We assessed the awareness of STMVs by asking respondents whether they had heard of at least one of the STMVs listed in the questionnaire. Other important derivers in the adoption of seed‐related technologies are related to the availability and affordability of the seed itself. We constructed a dummy variable for seed availability by asking respondents that were aware of the existence of STMVs but did not adopt them to give reasons for non‐adoption. Based on this question, we were able to identify two extra categories of households: (i) households that were aware of STMVs and that had physical seed access if they wanted to purchase, and (ii) households that were aware of the existence of STMVs and had affordable access to seed. The difference between the two groups is that the former focuses on the supply side of the seed, thus making seed available to the farmer while the latter is confounded by both the supply, and demand side, as farmers may fail to purchase seed even when it is availed to them at a price higher than they can afford. Out of 720 farmers in the sample, 27% were aware of STMVs, 21% had seed access (regardless of affordability), while 19% had seed access at a price they could afford.

^7^
The farmgate maize price used in the computation was based on prices offered by buyers in the study regions

^8^
We only report IV estimates where we considered the amount of land under STMV (scale of adoption) and the mere adoption (0,1) of STMV as endogenous. We used exposure to STMVs and household land size as instruments, in the binary adoption and adoption scale equations

## 1

**TABLE A1:** Determinants of adoption and of adoption intensity—Heckman bivariate probit model

Variable	Heckman selection model—two‐step estimates (regression model with sample selection)		
	Coef.	SE	Z
Treatment scale			
Seed (Kg/Ha)	−4.214***	1.081	−3.9
Fertilizer (Kg/Ha) (Ln)	0.261**	0.12	2.17
Fertilizer (Kg/ Ha) (sq)	−0.168	0.096	−1.75
Pesticide (Lit/Ha)	−0.056	0.106	−0.53
Herbicides (Lit/ Ha)	0.139	0.23	0.61
Organic fertilizer (Kg/Ha)	−0.07	0.076	−0.92
Soil fertile plots	−0.24	0.828	−0.29
No soil erosion plots	0.391	0.778	0.5
Plots with cover crop remains	2.661**	1.114	2.39
Household size (No.)	−1.844**	0.883	−2.09
Income allows to build savings	2.178*	1.244	1.75
Income allows to save very little	0.461	0.828	0.56
Group membership (1 = yes; 0 = otherwise)	−1.66*	0.86	−1.93
Farm size	6.364***	0.515	12.36
_constant	23.853***	4.045	5.9
Treatment (0,1)			
Seed (Kg/Ha)	−0.425***	0.129	−3.3
Fertilizer (Kg/Ha) (Ln)	0.003	0.016	0.2
Fertilizer (Kg/ Ha) (sq)	0.031**	0.013	2.41
Pesticide (Lit/Ha)	−0.009	0.016	−0.56
Herbicides (Lit/ Ha)	−0.006	0.032	−0.2
Organic fertilizer (Kg/Ha)	0.013	0.012	1.12
Soil fertile plots	0.251**	0.118	2.13
No soil erosion plots	−0.035	0.112	−0.31
Plots with cover crop remains	0.216	0.176	1.23
Household size (Ln)	0.132	0.118	1.12
Income able to build savings	−0.087	0.182	−0.48
Income able to save very little	0.166	0.116	1.43
Group membership (1 = Yes; 0 = Otherwise)	0.346***	0.114	3.04
Exposure to stress tolerant maize	1.059***	0.124	8.52
_constant	−0.685	0.516	−1.33
Lambda	0.503	1.643	0.31
Rho	0.078		
Sigma	6.458		

**TABLE A2:** Switch‐ probit model estimates of STMV adoption and food security

Variable	Determinants of STMV adoption (Adoption = 1)		Determinants of food security (Food secure = 1)			
			Adopters		Non‐adopters	
Adoptstma	Coeff	SE	Coeff	SE	Coeff	SE
Gender	0.112	0.149	−0.079	0.231	−0.251	0.166
Lnfarmsize	−0.003	0.071	−0.005	0.106	−0.022	0.079
Lnhhsize	0.046	0.119	−0.401**	0.183	−0.303**	0.125
Lndistancemkt	0.029	0.044	−0.044	0.062	−0.025	0.047
Credit	0.141***	0.040	0.042	0.081	−0.032	0.044
Nrooms	0.127	0.092	0.090	0.153	0.050	0.097
radio2	0.020	0.126	0.051	0.182	0.078	0.143
Mobile	−0.018	0.181	0.068	0.271	0.035	0.214
Tv	0.114	0.136	−0.088	0.191	0.110	0.148
Electrcharcoa	0.004	0.146	1.110***	0.328	0.423***	0.160
Mattress	−0.337**	0.158	0.877***	0.259	0.754***	0.216
lntotalincome2	0.013*	0.008	−0.009	0.012	−0.009	0.008
Cattle	0.016	0.012	−0.010	0.014	−0.006	0.014
Goats	0.014	0.009	0.020	0.016	−0.011	0.011
Poultry	0.001	0.001	0.006	0.004	0.007	0.005
Mbeya	−0.473**	0.185	0.088	0.389	0.127	0.186
Tabora	−0.192	0.166	0.368	0.240	−0.018	0.193
Manyara	0.148	0.158	0.283	0.242	0.109	0.180
Kilimanjaro	0.370**	0.155	0.211	0.261	−0.331*	0.172
Iringa	−0.544**	0.221	1.229***	0.431	0.384*	0.213
ReceivedSeedInfo	0.176**	0.086				
Lndistanceextn	0.056	0.046				
athrho1	−0.723	0.689				
athrho0	−13.123	1,535.791				
Constant	−1.067***	0.343	−0.384	1.041	−0.741**	0.377
Observations	703					
Wald test (*p *= 0)	80.74***	0.000				
Chi‐square test] = *‐+*/	80.74***	0.000				
Log likelihood	−843.65***					
rho0	−1		−1			
rho1	−0.619		−0.619			
p_c	0.0622		0.0622			
chi2_c	5.555		5.555			
ll_c	−846.4		−846.4			
k_aux	2		2			
